# Four cases of corneal perforation in patients with chronic graft-versus-host disease

**Published:** 2011-02-25

**Authors:** Emi Inagaki, Yoko Ogawa, Yukihiro Matsumoto, Tetsuya Kawakita, Shigeto Shimmura, Kazuo Tsubota

**Affiliations:** Department of Ophthalmology, Keio University School of Medicine, Tokyo, Japan

## Abstract

**Purpose:**

To report the clinical features and investigate the underlying pathological processes of spontaneous corneal perforation in patients with ocular chronic graft-versus-host disease (cGVHD).

**Methods:**

A full ophthalmological evaluation of corneal perforation in four patients with cGVHD was performed. Three of them underwent deep anterior lamellar keratoplasty and samples from two of three patients were used for histopathological analyses.

**Results:**

Three patients were successfully treated by corneal transplantation. One patient was treated with a therapeutic soft contact lens, and the wound healed within 2 days. The common clinical features of these patients were (1) the presence of definite dry eye related to cGVHD in 3 of 4 patients and probable dry eye in one patient, (2) a central or paracentral site of corneal ulceration and perforation, with no sign of infection, and (3) prior use of a topical or systemic corticosteroid, and/or topical non-steroidal anti-inflammatory drugs. Immunohistochemical findings revealed an increased number of cluster of differentiation 68^+^ (CD68^+^) macrophages and matrix metalloproteinase 9 (MMP-9) expression in the tissue surrounding the perforation.

**Conclusions:**

Our report extends current information on the clinical features and pathological processes of corneal perforation in cGVHD by showing increased MMP-9 expression and the accumulation of CD68^+^ positive macrophages in the affected areas.

## Introduction

Allogenic hematopoietic stem cell transplantation (HSCT) is now a curative therapy for several hematological malignancies and inherited disorders [1]. As a result, the number of HSCTs performed annually (about 25,000) has increased worldwide, as has the incidence of chronic graft-versus-host disease (cGVHD), a serious complication following HSCT [2]. The eye is a frequent target of cGVHD after HSCT, with the involvement of both the ocular surface and posterior ocular structures [3,4]. In particular, the effects of cGVHD on the cornea can cause irreversible damage, including corneal calcification, ulceration, thinning, and melting [5-7]. In patients with cGVHD after HSCT, corneal perforation is a serious condition that can lead to blindness. Several etiologies for corneal perforation in cGVHD have been proposed, including the presence of apoptotic cells, and immunological reactions in perforated cornea [8-11].

Here, we studied 4 cases of corneal perforation associated with cGVHD and described the patients’ clinical histories, analyzing common factors that could contribute to corneal perforation. We also described histopathological and immunohistochemical findings from cGVHD perforated corneal samples.

## Methods

Four eyes of 4 patients with ocular cGVHD (1 female, 3 males; median age, 59 years (range 21 to 70) and corneal perforation were studied. The type of HSCT was allogenic peripheral blood stem cell transplantation (PBSCT) in 2 cases and allogeneic bone marrow transplantation with a reduced-intensity preparative regimen in 2 cases. The diagnosis of corneal perforation was made by slit-lamp examination in combination with a Seidel’s test. We diagnosed dry eye when tear film instability was detectable (Tear break up time ≤5 s and/or Schirmer test ≤5 mm) and any abnormality of ocular surface (Rose Bengal score ≥3 and/or fluorescein staining score ≥3) and symptoms of ocular irritation. Definite dry eye was defined as having all three components including tear film instability, abnormality of ocular surface and, symptoms of ocular irritation. Probable dry eye was defined as having one of either tear film instability or abnormality of ocular surface along with symptom of ocular irritation [12]. Meibomian gland dysfunction (MGD) was scored by Shimazaki’s grading system [13]. The assessment of obstruction of the meibomian gland orfice was conducted by digital pressure applied on the upper tarsus, and the degree of ease in expressing meibomian secretion (meibum) was evaluated semi-quantitatively as follows; grade 0, clear meibum is easily expressed; grade 1, clear meibum is expressed with more than moderate pressure; grade 2, cloudy meibum is expressed with more than moderate pressure; and grade 3, meibum cannot be expressed even with the hard pressure.

All four patients fulfilled the National Institutes of Health (NIH, Bethesda, MD) consensus criteria for a diagnosis of cGVHD [14], which was at least 1 diagnostic clinical sign of cGVHD or the presence of at least 1 distinctive cGVHD manifestation (e.g., keratoconjunctivitis sicca) confirmed by pertinent biopsy or other relevant tests (e.g., Schirmer test) in the same or another organ. The time from HSCT to corneal perforation, from the onset of corneal ulcer to corneal perforation, and from HSCT to the onset of dry eye were determined. We also analyzed the position and size of the corneal ulcers and corneal perforation and the degree of dry eye. We examined samples of perforated corneal tissue obtained during keratoplasty. We could not obtain a sample of perforated corneal tissue from one of the three patients during surgery. Therefore, we performed histological studies on samples from only two patients. Eye bank corneas from overseas were used as controls.

Our research protocols followed the tenets of the Declaration of Helsinki, and informed consent was obtained from all the subjects after an explanation of the nature and possible consequences of the study. We obtained approval for this study from the IRB/Ethics committee at Keio University.

A portion of each corneal specimen was immediately embedded in Tissue-Tek O.C.T. compound (Miles Inc., Elkhart, IN) and stored at –80 °C until sectioning. The remainder of each sample was fixed in 10% neutral buffered formalin, embedded in paraffin wax, processed according to conventional histologic techniques, and stained with hematoxylin-eosin. Frozen sections 5-μm thick were used for immunohistochemical analyses, which were performed according to a standard protocol [15,16]. Briefly, the sections were blocked with 10% goat serum for 1 h and then were incubated a panel of mouse monoclonal antibodies against cluster of differntiation 4^+^ (CD4; RPA-T4; Biolegend, San Diego, CA), cluster of differentiation 8^+^ (CD8; C8/144B; Nichirei Bioscience, Tokyo, Japan), and cluster of differentiation 68^+^ (CD68^+^; Dako, Glostrup, Denmark), and a peroxidase-conjugated rabbit anti-mouse secondary IgG (Dako). Matrix Metalloproteinase-2 (MMP2) was labeled with a rabbit anti-human MMP2 antibody (Daiichi Fine Chemical Co., LTD., Toyama, Japan), and MMP9 with a mouse anti-human MMP9 antibody (Dako) [15,16]. The secondary antibodes for MMP2 and MMP9 were Alexa 488-conjugated goat anti-mouse (Molecular Probes, Eugene, OR). Nuclei were counterstained with TO-PRO-3 (Molecular Probes). Isotype-matched fluorescein-conjugated mouse antibodies were used as controls. The sections were examined with an ECLIPSE E800 microscope (Nikon, Tokyo, Japan) or a LSM5 PASCAL 510 confocal microscope (Carl-Zeiss, Göttingen, Germany).

## Results

### Clinical manifestations in 4 cases of corneal perforation associated with cGVHD

The clinical characteristics of the 4 patients with cGVHD are shown in Table 1 and Table 2. Three of 4 patients had definite dry eye, and one patient had probable dry eye, before the onset of corneal ulcer and perforation. The mean onset of dry eye related to cGVHD in 3 of 4 patients was 4.7±1.2 months after HSCT, which is typical for this condition. Severe obstruction of meibomian gland orfice (grade 3), entropion, and corneal neovascularization were observed in 2 of the 4 cases. The mean onset of corneal perforation after HSCT for all four cases was 23.8±8.1 months. The time from the diagnosis of corneal ulcer to perforation was 1 to 5 weeks. The mean time from the onset of dry eye to corneal perforation was 17.7±7.6 months. The corneal perforations were all central or paracentral (Table 2), and there was no indication of infection in any of the cases. Conjunctival hyperemia and corneal opacity were not prominent in these samples of cGVHD-associated corneal perforation, although these findings are frequent in cases of Sjögren’s syndrome and infection. The size of perforations was relatively small, ranging from 0.5×1.0 mm to 0.5×2.0 mm. Three patients were treated with deep anterior lamellar keratoplasty (DALK). The fourth patient was treated successfully by using therapeutic soft contact lenses, and no surgery was required (Table 2). Following treatment, all the patients showed a stable ocular surface condition.

**Table 1:** Clinical characteristics of chronic graft versus host disease patients with corneal perforation.

**Case number**	**Case 1**	**Case 2**	**Case 3**	**Case 4**
Age (Years), sex	62, M	21, M	70, M	56, F
Underlying disease	AML (M4)	AML (M0)	AML (M4)	CML
Type of treatment	Allo-PBSCT	mini BMT	Allo-PBSCT	mini BMT
Onset of dry eye (month)	6	4	4	N.A.
Onset of corneal perforation (month)	17	20	30	8
Schirmer Test (mm)	1	0	4	10
Fluorescein Score (9 points)	9	9	3	3
MGD score (3 points)	Unknown	3	3	Unknown
External bacterial flora culture	MRSA	Trichosporon spp.	MRSA	negative
Entropion	−	+	+	−
Corneal neovascularization	−	+	+	−
Topical drops (corticosteroid)	+ (1 day)*	+ (5 years)**	+ (3 weeks)***	−
Topical drops (NSAIDS)	−	−	+ (3 weeks)§	+ (4 months)¶
Oral corticosteroids (PSL)	+ (3 months)	+ (15 mg) (6 months)	+ (10 mg) (> 2 years)	+ (> 20 mg) (2 months)

AML, acute myeloid leukemia; CML, chronic myeloid leukemia; allo-PBSCT, allogeneic peripheral blood stem cell transplantation; MGD, meibomian gland dysfunction; MRSA, methicillin-resistant *Staphylococcus aureus*; mini BMT, mini bone marrow transplantation, reduced intensity of conditioning regimen for hematopoietic stem cell transplantation; NSAIDS, non-steroidal anti-inflammatory drugs; *, dexamethasone sodium phosphate; **, 0.02% fluorometholone; ***, 0.1% fluorometholone; §, dicrofenac sodium;¶, bromfenac sodium, Topical corticosteroids were used 4 times per day; Topical NSAIDS were used 2 times per day. The dose (per day) and duration of oral corticosteroid were shown in the table. PSL, predonisolone; mo, months.

**Table 2:** Clinical findings of corneal ulcer and perforation.

**Case number**	**Case 1**	**Case 2**	**Case 3**	**Case 4**
Size of corneal ulcer (mm)	2.0×1.5	2.0×2.5	2.0×1.5	3.5×1.5
Position of corneal ulcer	center	paracenter	paracenter	paracenter
Size of corneal perforation (mm)	0.5×0.5	0.5×1.0	0.5×0.5	2.0×1.0
Position of corneal perforation	center	paracenter	paracenter	paracenter
Period from corneal ulcer to perforation (day)	7	12	within 35	within 27
Preoperative visual acuity	20/100	LP (+)	20/250	20/250
Surgical intervention	glue, DALK	DALK	MUCL	DALK
Postoperative visual acuity	20/200	20/250	20/50	20/500
Observation periods (month)	7	2	40	15

MUCL, medical use contact lens ; DALK, deep anterior lamellar keratoplasty.

#### Case 1

**Figure 1:**
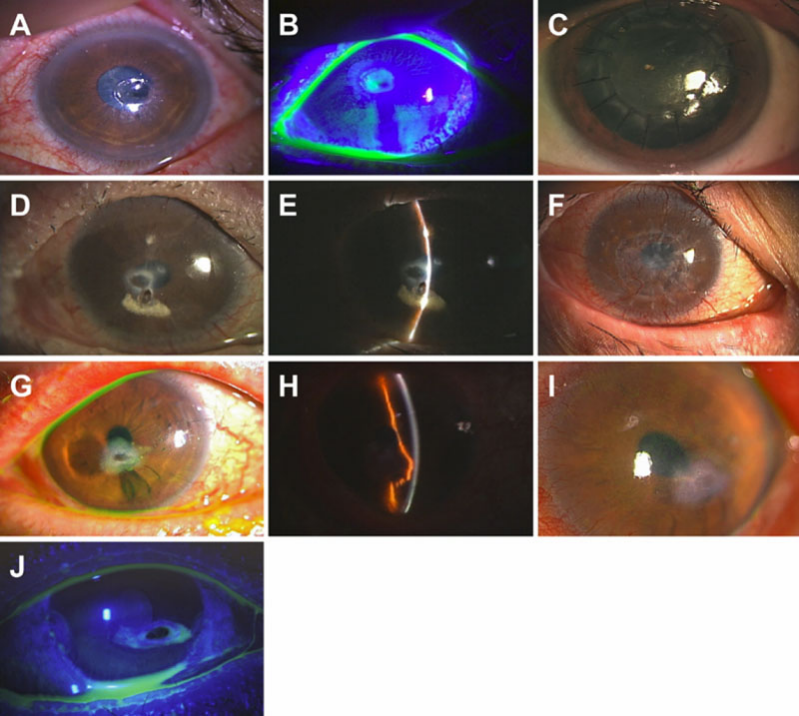
Clinical findings of perforated cornea before and after treatment with deep anterior lamellar keratoplasty or glue. **A**, **B**: Case 1 photographs of the perforated cornea upon admission (left eye). **A**: The perforation was located in the paracentral cornea. The size of the perforation was 0.5 mm×0.5 mm. The surrounding ulcer was 2.0 mm×1.5 mm. **B**: Preoperative photo showing a positive Seidel test. **C**: Photograph of the cornea after deep anterior lamellar keratoplasty. Five months later, the corneal graft remained stable and transparent. **D**, **E**: Case 2 slit lamp photograph taken upon admission (right eye). **D**: The perforation was in the paracentral cornea and was 0.5 mm×1.0 mm. **E**: Photograph showing the paracentral corneal perforation plugged by the iris. **F**: Slit lamp photograph after the operation. One year after deep anterior lamellar keratoplasty, the corneal graft was stable and transparent. **G**, **H**: Case 3 slit lamp photograph taken upon admission (left eye). **G**: The perforation was inferior to the center of the cornea and was 0.5 mm×0.5 mm. An ulcer (2.0 mm×1.0 mm) was observed inferiorly. **H**: The anterior chamber was maintained by the prolapsed iris. **I**: Slit lamp photograph 3 months after healing of the perforated cornea without surgical intervention. **J**: Case 4 slit lamp photograph taken upon admission (right eye). The perforation was located inferior to the center of the cornea. The cornea was 3.5 mm×1.5 mm, and the perforation was 2.0 mm×1.0 mm. The Seidel test was positive.

A 62-year-old man had been treated for acute myelogenous leukemia (AML; M4) with an allogeneic peripheral blood stem cell transplant from an human leukocyte antigen (HLA)-matched sibling in April, 2006. Six months later, he presented with severe dry eye related to cGVHD. The dry eye was treated at a local eye clinic with commercially available tear substitutes and punctal plug. In August, 2007, the subject exhibited a grouped vesicular eruption over dermatome V1 on his right forehead. Five days later, he developed superficial punctate keratitis, and uveitis in the right eye, and corneal erosion in the left eye. He was treated with topical eye medications that contained acyclovir (Santen Pharmaceutical Co. Ltd., Osaka, Japan), sodium hyaluronate, (Santen Pharmaceurical Co. Ltd, Osaka, Japan) and betamethasone sodium phosphate (Shionogi & Co., Ltd., Osaka, Japan) for both eyes and gatifloxacin hydrate (Senju Pharmaceutical Co., Ltd., Osaka, Japan) and 1% atropine sulfate (Nitten Pharmaceutical Co., Ltd., Nagoya, Japan) for the right eye. However, after 1 day of this treatment, the patient developed a central corneal perforation of the left eye (Figure 1A,B). Cyanoacrylate glue therapy was attempted, but failed on the following day. The perforation was then treated by DALK. Best-corrected visual acuity (BCVA) at the 6-month follow up was 20/200. The corneal graft attached well and was stable (Figure 1C).

#### Case 2

A 20-year-old man had been treated for AML (M0) with allogeneic peripheral blood stem cell transplantation (PBSCT) in March, 2005. Two months later, severe cGVHD-related dry eye was diagnosed and treated with tear substitutes, including eyedrops containing autologous sera and 0.1% topical sodium hyaluronate, 0.02% fluorometholone (Santen Pharmaceurical Co. Ltd.) and levofloxacin (Santen Pharmaceurical Co. Ltd.). Eight months after the diagnosis of severe dry eye, he was referred to our hospital with epiphora from his left eye, and slit lamp examination revealed a perforation in the center of the cornea (Figure 1D,E). BCVA on his first visit was light perception in the left eye. He underwent DALK. BCVA at the 2-year follow up was 20/200. The graft remained clear and no recurrence was observed (Figure 1F).

#### Case 3

A 70-year-old man had been treated for AML (M4) with allogeneic PBSCT in April, 2002. Two years later, he experienced mild dry eye with superficial punctate keratitis in both eyes. Four months after the onset of dry eye, he underwent cataract surgery of both eyes, and his post-surgical treatment included topical non-steroidal anti-inflammatory eye drops (topical 0.1% diclofenac sodium; Wakamoto Co., Ltd, Tokyo, Japan) and topical 0.1% fluorometholone. Three weeks after the cataract surgery, corneal perforation was diagnosed at a local clinic, and the patient was referred to our hospital. The perforation was inferior to the center of the cornea on the left eye (Figure 1G). An ulcer was observed inferior to the perforation. The anterior chamber was maintained by the incarcerated prolapsed iris (Figure 1H). BCVA upon presentation was 20/200 in the left eye. He was treated with a therapeutic contact lens, and the wound had healed by the second day after admission. On the 5th day, re-epithelization was evident. Three months later, a slit lamp examination showed that the perforated cornea had healed without surgical intervention (Figure 1I). BCVA at the 3-year follow up was 20/50.

#### Case 4

A 56-year-old female patient had been treated for AML with allogenic PBSCT from an HLA-identical sibling after a reduced-intensity preparative regimen, in July, 2002. Five months later, she had dry eye sensation and painful eye with full areas of punctate keratopathy and liver cGVHD. Probable dry eye related to cGVHD was present and treated with tear substitutes and topical nonsteroidal anti-inflammatory eye drops (bromfenac sodium hydrate eye drops; Senju Pharmaceutical co, Ltd, Osaka, Japan). Sytemic cGVHD was treated with an oral predonisolone. Nine months after HSCT, she presented with epiphora, a paracentral corneal perforation of her right eye was diagnosed (Figure 1J), and she was referred to our hospital for treatment. BCVA upon presentation was 20/200 in the right eye. An ulcer was observed inferior to the perforation. The perforated cornea was treated with DALK. A slit lamp examination 5 months after the surgery showed a clear and stable corneal graft. BCVA at the 1-year follow up was 20/200.

### Histopathologic findings

**Figure 2:**
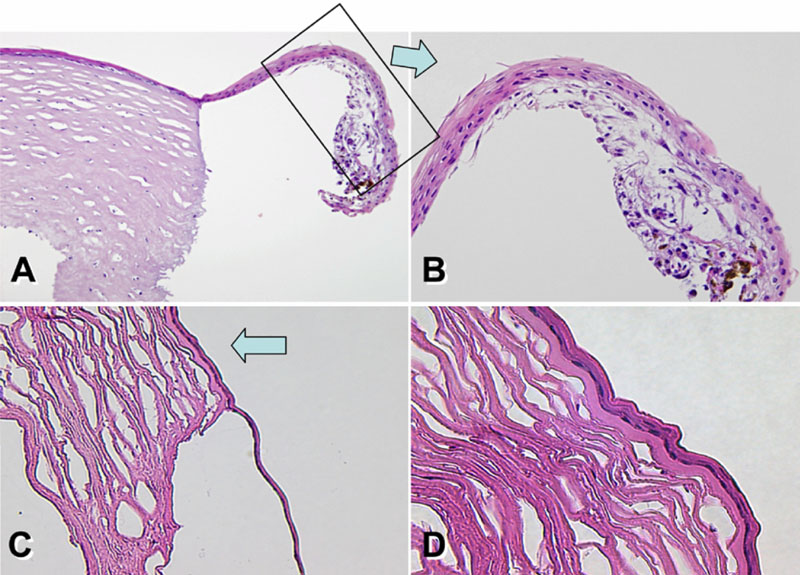
Pathological findings. **A**, **B**: Case 4 frozen tissue section (cornea, hematoxylin & eosin staining, original magnification 40×, 100×). **B**: Magnified view of boxed area with an arrow in **A**. Corneal tissue near the perforation. **C**, **D**: Frozen tissue section (cornea, hematoxylin & eosin staining, original magnification 100×, 200×) Corneal tissue near the perforation. The epithelium neighboring the corneal ulcer showed partial thinning (arrow).

To investigate the histopathology of cGVHD-associated corneal perforation, we used samples from case patients 1 and 4, obtained during DALK. Hematoxylin and eosin staining of both perforated corneal samples revealed almost no neutrophilic infiltration in the stroma near the corneal ulcer. Melting of the corneal stroma was observed locally; however, the rest of the stromal tissue remained intact. The epithelium neighboring the corneal ulcer showed partial thinning (Figure 2A,B; patient 4) compared with normal control corneal tissue (data not shown). Similar findings were observe in the perforated cornea obtained from another patient. (Figure 2C,D; patient 1).

### Immunohistochemistry

**Figure 3:**
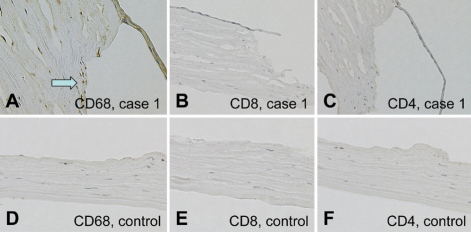
Immunohistochemical findings (CD4, CD8, and CD68). **A**: CD68 expression at the margin of the corneal ulcer from case 4 sample. **B**, **C**: CD8^+^ (**B**) and CD4^+^ (**C**) T cells were not detected in this sample. **E**, **F**: Control samples for CD8 (**E**) and CD4 (**F**) immunostaining. **D**: Control sample. CD68 immunostaining.

**Figure 4:**
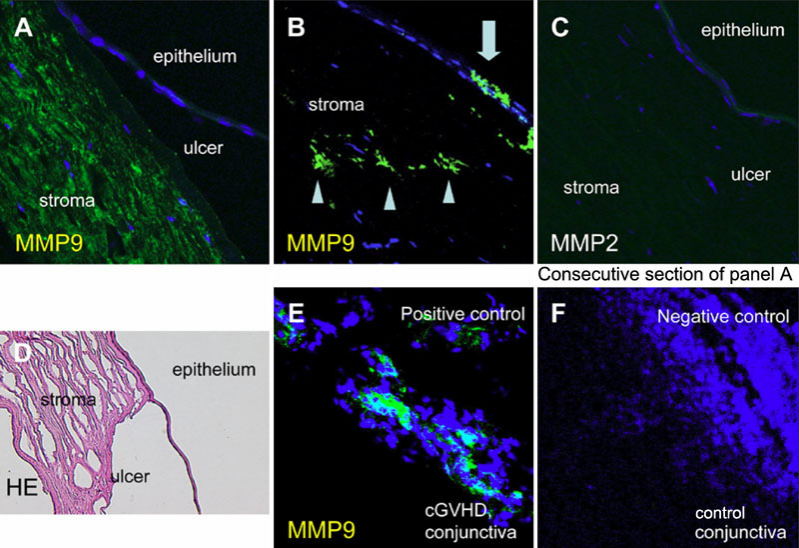
Immunohistochemical findings (MMP9 and MMP2). **A**-**D**: Consecutive sections from the case 1 sample. **A**, **B**: Intense MMP9 expression was seen in the stroma (**A**, **B**) and epithelium (**B**) near the perforation. **C**: MMP2 expression was not observed. **E**: Conjunctiva sample from a cGVHD patient showing intense MMP9 expression (positive control). **F**: Control conjunctiva sample (negative control).

Staining for CD68 showed intense expression on mononuclear cells around the margin of the corneal ulcer in the sample from case 1 (Figure 3A), but scarcely detected in the control samples (Figure 3D). Both CD8^+^ (Figure 3B,E) and CD4^+^ (Figure 3C,F) T cells were not detected in these samples or control samles. Immunostaining for MMP9, also known as type IV collagenase, showed intense expression in the stroma (Figure 4A,B) and epithelium (Figure 4B) of the perforated cornea from a patient with cGVHD. However, no immunoreactivity for MMP2 was detected (Figure 4C). MMP9 was not detected in the negative control samples (Figure 4F).

## Discussion

In this study, we individually described the clinical characteristics of four patients with corneal perforation. As common factors in our cases of cGVHD-associated perforated cornea, we noted definite dry eye related to cGVHD in 3 of 4 patients and one patient had new onset of probable dry eye related to cGVHD and the use of topical or systemic corticosteroids and/or topical NSAIDS before the corneal perforation. In addition, in all of the cases, the corneal ulcer and perforation occurred centrally or paracentrally with no sign of infection or neutrophilic infiltration. We also performed histologic and immunohistologic examinations of perforated cornea tissue samples from two patients. To our knowledge, we have shown for the first time MMP9 expression and CD68^+^ macrophages accumulation in the tissue surounding the perforation which may contribute to corneal perforation in patients with cGVHD.

Dry eye associated with cGVHD is a major late complication after HSCT [3,4,17]. The new onset of dry eye, gritty and painful eyes, and cicatricial conjunctivitis are distinctive features of ocular cGVHD [14]. Once dry eye develops, even more severe dryness toward the center of the cornea can easily occur. Dry eye related to cGVHD is often associated with conjunctival fibrosis, trichiasis due to cicatricial changes, and limbal stem cell deficiency, which leads to corneal neovascularization [18,19].

In Sjögren's syndrome, corneal ulceration and perforation associated with topical corticosteroid use have been reported [20]. The authors of that study strongly recommend avoiding topical steroid use, to prevent corneal perforation. The previous reports in Sjögren's syndrome suggest us to avoid excessive topical corticosteroids use, particularly because their systemic use is necessary for HSCT recipients to prevent or treat cGVHD. It is also necessary to avoid ocular complications of corticosteroid use, such as cataract, glaucoma, and infection in HSCT recipients. This policy may also reduce the risk of corneal perforation. It has been also reported that the use of topical nonsteroidal anti-inflammatory eyedrops after ocular surgery is associated with corneal melting [21,22]. Topical eyedrops might be a culprit for the development of corneal perforation in cGVHD.

The corneal perforations in this case series were all paracentral or central. This localization may be resembling neurotrophic ulcer and limbal stem cell deficiency, which are frequent features of chronic inflammation [23,24]. The idea that inflammatory or immune responses could increase the risk of corneal perforation in cGVHD is consistent with reports of corneal perforation in autoimmune diseases like rheumatoid arthritis and Sjögren’s syndrome, and in corneal infections. Interestingly, these conditions also result in paracentral perforations, as in our series [25-29].

**Figure 5:**
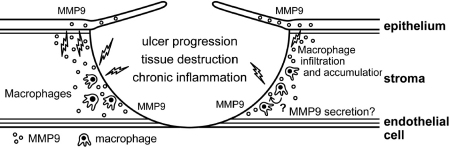
Hypothetical etiology of corneal perforation in cGVHD patients. CD68^+^ macrophages engulf and degrade the recipient’s degenerated epithelium as a foreign body, leading to the secretion of a large amount of proinflammatory cytokines, and the subsequent expression of MMP9 on macrophages or the corneal stroma and epithelium.

In addition, sections through the corneal perforation site of our cases 1 and 4 showed an increased infiltration of CD68^+^ macrophages and immunostaining for MMP9. The accumulation of CD68^+^ macrophages at the site of corneal perforation reflects local immune responses, including the inflammatory response. CD68^+^ macrophages might engulf and destroy the recipient’s damaged corneal tissues, leading to the release of large amounts of pro-inflammatory cytokines and inducing the expression of MMPs (Figure 5), but it is not clear whether the CD68^+^ macrophage infiltration is a primary event, which may induce corneal perforation, or a response to it. Further study is needed to clarify this issue.

MMPs participate in extracellular matrix remodeling during acute and chronic inflammation. They regulate the inflammatory cascade by acting on inflammatory mediators and their receptors [30]. Several studies suggest a relationship between MMP9 expression and dry eye or other ocular diseases, including recurrent corneal melting in patients with primary Sjögren’s syndrome [25] and melted corneal grafts [31]. Overexpression of MMP2 in the cornea and the presence of MMP9 in the tear film have been found in cases of peripheral ulcerative keratitis [32]. Thus, MMP9 may be involved in the pathology that led to corneal perforation in our series of patients. MMP9 can be expressed by macrophages, the corneal epithelium, extracellular matrix, and/or in response to cell-cell interactions; in contrast, only keratocytes secrete MMP2 in the eye [24]. In any case, understanding the mechanisms resulting in dry eye, inflammatory cell infiltration into the cornea, and the pattern of MMP9 expression in the corneal microenvironment will help to clarify some of the processes leading to corneal perforation in cGVHD patients. The blockade of MMP9 expression and inhibition of macrophage infiltration may be useful for preventing corneal perforation in these patients.

Furthermore, Suzuki et al. [10] reported that CD8^+^ T cells infiltrating the perforated cornea play an important role in the perforation, and strongly suggested that corneal perforation in ocular cGVHD results from CD8^+^ T cell-mediated cytotoxicity an idea that is compatible with cGVHD pathology. These findings fit with cGVHD’s similarity to an autoimmune disease, that is, as a disease of dysregulated immunity with multiple manifestations [33]. Finally, mechanical stress caused by conjunctival fibrosis or the pre-HSCT preparative regimen, which includes total body irradiation and/or massive chemotherapy, could influence the maintenance of the ocular surface.

Case control studies will be needed to identify clinical factors that might elevate the risk of corneal perforation and obtain more insight into the pathological processes that lead to corneal perforation in patients with cGVHD following allogeneic HSCT. However, our study and previous reports have several implications for clinical practice. Patients with ocular cGVHD who are treated with topical steroids or NSAIDS should be monitored closely due to the possible risk of corneal perforation and the potential for subsequent blindness. Understanding how patients with ocular cGVHD can avoid conditions that threaten their vision has become increasingly important with the success of HSCT and the long-term survival of greater numbers of patients. Our findings may help reduce the risk of corneal perforation in some patients with ocular cGVHD.
